# Chloroform Fatalities.—II

**Published:** 1895-07-13

**Authors:** Benjamin Ward Richardson


### Chloroform Fatalities.—II.

By Sir Benjamin Ward Richardson, M.D., F.R.S.

There is a remaining reform in regard to the employment of anæsthetics which ought at once to be carried out, a reform rather social than scientific. We started anæsthesia, as I well remember, with a bad hypothesis, and fashion founded on it. In the beginning, in all operations, the surgeon had the social lead, and he willed, and still wills, to keep it. The operation, be it ever so simple, safe, or light, something that the most ordinary surgeon could perform if shown the way, was held to stand before the anæsthesia. Not once in a hundred times might there be danger from the operation; every time there was danger from the anæsthetic; but it signified not, the operation took the first place.

In this manner the impression got abroad that anyone could administer chloroform, and for many years, to Snow's great indignation, a porter, or door-keeper, at one of the great hospitals was the chosen instrument. In other hospitals the youngest house surgeon, or clinical clerk, was the administrator, and when things changed in this direction, and regular specialists rose to do the duty, they remained as appendages to the surgical deity, who might be years younger and less experienced than themselves; a position entirely false.

I remember that Snow, being on one occasion about to administer chloroform to a lady of the upper class, took out his stethoscope to listen to the heart of his patient, as the pulse was irregular. To his surprise, a sister of the patient, standing by, pushed the stethoscope rather rudely aside, exclaiming as she did so, "What does this mean? If my sister has anything wrong with her chest, why is not Williams sent for?"—and it required all Ferguson's tact to explain that a chloroformist had "to try the heart as a particular test, before he began to administer." I remember another time when the footman who showed Dr. Snow into the drawing-room, after inquiring his business, announced him as "the person who gives the chloroform." The fees also for the so-called anæsthetist have been usually left with the surgeon as a part of the operation. Now, this is all wrong; a position resting on a false basis. If we had an anæsthetic that was absolutely safe, that never killed, an assistant, a nurse, or anyone at hand might be entrusted with the duty of administering; but we have not yet reached; that wonderful standard of precision and safety. The induction of anæsthesia is as yet an improving art, an art fraught with danger; an art requiring the most consummate skill, courage, and responsibility from the practitioner who carries it out. In all fairness such a man should hold his true place as the man upon whom multitudes depend for safety, and if he be not the captain in general command, he should be the pilot in dangerous passages, and maintain his position independently. Let him have time to study his cases at leisure, instead of being called upon as a mere machine to do instantly what he is bidden, and let him name his own fees for his task, quite apart from the operation or the surgeon who performs it.

I know this is delicate ground, but where great professional interests are at stake let us, as Goodsir used to say, "let us have the truth, the whole truth, and nothing but the truth."

The avoidance of accidents arising from general anæsthesia is of itself a problem. For the moment successful anæsthesia rests on skill, and danger would, there is no doubt, be considerably diminished by the selection of, and adherence to, a mode of administration that is uniform in its character, precise in its nature, and grounded on the knowledge of getting the best results from the smallest possible amount of the anæsthetic vapour. If the living body be saturated with the vapour; if more vapour be introduced than is necessary, more than the statutory 18 minims of chloroform to the whole volume of blood; if the blood have forced into it more than it can carry in solution; if the vascular organs become surcharged, then there are two dangers ahead, one of globules of the liquid obstructing the vessels of the minute circulation, acting, in fact, as so many gaseous plugs, like air in the veins; the other, of interference with the functions of organs, and the check to that oxidation which is essential to life; a check that chloroform has the power to exercise. These are dangers of a late stage, but often before they can be developed, dangers of reflex irritation, belonging to the first stage, immediately occur.

The sanguine are of opinion that by great skill in administration, by limiting the amount of chloroform used, by administering slowly—much in the same way as Dr. Snow did—by varying the process so as to adapt it to the seasons, and by making careful diagnosis, they will succeed in reducing the mortality to a minimum sufficiently satisfactory to the most critical. I do not think myself that any mode of administration will always meet the difficulty of the *morituri.* There will be occasional cases in which the mind so influences the body that fear itself will be a constant cause of death. I have seen syncope take place even under local anæsthesia, although pain was entirely removed. Sir William Ferguson once brought to me a clergyman from whom he wished to remove a loose tumour from what is called the ball of the thumb. He had formed the idea that the patient would not be a good subject for chloroform, and as the incision to be made was very superficial, and as he thought the tumour would roll out quite easily, it struck him that to freeze the part with spray would be all that was required. In a way the result was successful; the part in the line of incision was insensible within a minute; with exquisite skill he incised and rolled out the tumour—not a vessel had to be tied, and two stitches merely were required to close the wound. As the first of these stitches was being inserted Sir William called my attention to the patient, who was in a dead faint in the chair. We laid him on the floor, and, for the moment, thought that he was dead. Fortunately, under the restorative measures of recumbency, friction, and ammonia, the gentleman gradually came round; after which, thinking he had fainted from pain, I expressed my regret that the local anæsthesia had failed. "Not at all," he replied; "not at all. I felt nothing whatever; it was the glimmer of the knife that knocked me over." The recovery was complete; but that gentleman was amongst the *morituri*, and we both felt that if he had taken chloroform, and had fainted as he came under its influence, the syncope would have been fatal.

